# A Method for In-Vivo Mapping of Axonal Diameter Distributions in the Human Brain Using Diffusion-Based Axonal Spectrum Imaging (AxSI)

**DOI:** 10.1007/s12021-023-09630-w

**Published:** 2023-04-10

**Authors:** Hila Gast, Assaf Horowitz, Ronnie Krupnik, Daniel Barazany, Shlomi Lifshits, Shani Ben-Amitay, Yaniv Assaf

**Affiliations:** 1 Sagol School of Neuroscience, Tel Aviv University, Tel Aviv, Israel; 2 The Strauss center for neuroimaging, Tel Aviv University, Tel Aviv, Israel; 3 Department of Statistics and Operations Research, Faculty of Exact Sciences, Tel Aviv University, Tel-Aviv, Israel; 4 School of Neurobiology, Biochemistry and Biophysics, Faculty of Life Sciences, Tel Aviv University, Tel Aviv, Israel

**Keywords:** Brain MRI, Diffusion MRI, Axon diameter estimation, HCP, Image analysis, Axonal spectrum imaging

## Abstract

In this paper we demonstrate a generalized and simplified pipeline called axonal spectrum imaging (AxSI) for *in-vivo* estimation of axonal characteristics in the human brain. Whole-brain estimation of the axon diameter, *in-vivo* and *non-invasively*, across all fiber systems will allow exploring uncharted aspects of brain structure and function relations with emphasis on connectivity and connectome analysis. While axon diameter mapping is important in and of itself, its correlation with conduction velocity will allow, for the first time, the explorations of information transfer mechanisms within the brain. We demonstrate various well-known aspects of axonal morphometry (e.g., the corpus callosum axon diameter variation) as well as other aspects that are less explored (e.g., axon diameter-based separation of the superior longitudinal fasciculus into segments). Moreover, we have created an MNI based mean axon diameter map over the entire brain for a large cohort of subjects providing the reference basis for future studies exploring relation between axon properties, its connectome representation, and other functional and behavioral aspects of the brain.

**Supplementary Information:**

The online version contains supplementary material available at 10.1007/s12021-023-09630-w.

## Introduction

The ability to measure physiological features of the nervous system in humans is limited. Classical approaches such as single unit recording, electrocorticography (eCog) or local field potentials can be measured in humans invasively when aligned with other clinical needs (Engel et al., 2005; Parvizi & Kastner, 2018). The investigation of healthy brain neurophysiology is therefore confounded to non-invasive techniques such as evoked potentials (via EEG) and functional imaging (via BOLD fMRI or fNIRS) (Toga, 2015). While these methods have tremendously affected our knowledge on the functioning human brain, each one lacks important features such as temporal or spatial resolution, as well as specificity. Consequently, when diffusion MRI was shown to be sensitive to axonal morphology, it potentially provided an opportunity to estimate, for the first time, *in-vivo* and non-invasively, the microstructural property of axon diameter. Being meaningful by itself, it also provided a lead to explore one of the most basic physiological features of the brain - the conduction velocity, as conduction velocity was shown to be correlated with axon diameter (Gasser & Grundfest, 1939; Goldman & Albus, 1968; Horowitz et al., 2015; Waxman, 1980).

The Diffusion MRI signal has been shown to be sensitive to axon diameter under unique experimental conditions (Assaf et al., 2008, 2013, 2019; Barazany et al., 2009). Several biophysical models were developed to estimate the axonal diameter distribution from the diffusion MRI signal: the first was AxCaliber, followed by ActiveAx, AxCaliber3D, ActiveAxADD, Commit, Amico, and other, unnamed, methods (Barakovic et al., 2021; Barazany et al., 2011; Daducci et al., 2015; Drakesmith et al., 2019; Dyrby et al., 2013; Harkins et al., 2021; Rasclosa et al., 2015; Romascano et al., 2020; Veraart et al., 2020). These models demonstrated their usefulness for several distinct tracts/bundles mainly focusing on the corpus callosum. This sensitivity towards axon diameter was validated versus the state-of-the-art histology (electron microscopy) (Barazany et al., 2009; Harkins et al., 2021; Lee et al., 2019; Sepehrband et al., 2016). While first indications of diffusion MRI sensitivity towards axon diameter appeared to be very promising, following works raised concerns regarding the sensitivity, specificity, and suitability of this approach for exploring axon diameters in a clinical setup.

The ability to infer information of axon diameter is embedded in the assumption that diffusion within axons is restricted while elsewhere it is free or hindered. In an axon diameter sensitive diffusion MRI experiment, we need to favor the contribution of restricted diffusion over other modes of diffusion (Assaf et al., 2008; Huang et al., 2015, 2020). This might be achieved by tuning of the experimental parameters (i.e., the diffusion times (Δ) and diffusion weighting (b-value)) (Assaf & Cohen, 1998, 2000; Beaulieu, 2002; Beaulieu et al., 1998).

Several studies challenged the assumed sensitivity, specificity and selectivity of restricted diffusion towards intra-axonal water (Lee et al., 2018, 2020; Yoshiura et al., 2001). While they may point to valid concerns, experimental evidences suggest that the contribution of intra-axonal water to restricted diffusion signal dominates other factors (see Online Resource 1, section A) (Assaf et al., 2002; Assaf & Cohen, 1998, 2000; Beaulieu, 2002; Beaulieu et al., 1998; Peled et al., 1999; Stanisz et al., 1997; Stanisz & Henkelman, 1998) leading to the following conclusions:

1. Myelin causes significant restricted diffusion in neuronal tissue.
2. Diffusion experiments at high b-values (> 1000 s/mm^2^) are selective and specific to restricted diffusion water pools.
3. Intra-axonal water is the main source of restricted diffusion water population in neuronal tissue.

These evidences were the basis for axon diameter estimation frameworks (as listed above). In all these frameworks, the tissue is modeled as a combination of hindered and free diffusion in the extra-axonal space and diffusion within impermeable cylinders to represent the intra-axonal space. The models differ in their estimation of fiber orientation dispersion, exchange rates between the modeled compartments, as well as in the analytical description of diffusion within cylinders.

Assuming that certain experimental conditions indeed favor intra-axonal water signal, other critiques were raised whether these experimental conditions can be met in a clinical MRI setup (Veraart et al., 2020, 2021; Yoshiura et al., 2001) or even infeasible at all (Paquette et al., 2021). However, experiments designed to tackle these concerns suggested that they are not as severe as suspected (see Online Resource 1, section B). Noteworthy and most important is the invariance of the signal decay to the gradient duration when measured *in-vivo*. This invariance paves the path to the potential suitability of clinical scanners to axon diameter estimation through diffusion imaging. Moreover, it also greatly simplifies the modeling approach leading to an easy to implement and robust modeling framework termed AxSI.

The AxSI (Axonal Spectrum Imaging) framework, introduced in this paper, suggests a simplified approach to estimate axon diameter distribution per reconstructed axonal fiber or fascicle streamline (see Methods) dealing with all the above-mentioned concerns. Validation of this method on rodent data (see Online Resource 1, section C) indicates the high applicability and suitability of this method to a clinical MRI setup. It must be noted that the accurate, exact, and absolute calculation of the axon diameter is most likely unachievable, yet we claim that AxSI provides an accessible proxy of axon diameter information. We demonstrate the outcome of this generalized framework for the estimation of axon diameter properties of several fiber systems in the human brain, including but not restricted to the corpus callosum. We further show the utility of using AxSI for estimating ADD over a large database of diffusion MRI measurements serving as a reference base for future studies on conduction velocity and brain function and behavior. Specifically, we demonstrate the ability to use AxSI for connectome analysis, incorporating axon diameter as indicator of information transfer speed.

### AxSI Modeling Framework

In this paper we suggest a framework for estimating axonal diameter distributions, per extracted fiber streamline, called axonal spectrum imaging (AxSI). AxSI uses several experimental considerations that simplify both acquisition and modeling algorithms on one hand and overfitting by many free parameters on the other hand.

AxSI follows the same general description of AxCaliber (and other frameworks) that suggest that the measured signal is a linear combination of three water pools (Assaf et al., 2004; Assaf & Basser, 2005): CSF water, hindered diffusion (cellular and extracellular) and restricted diffusion (axonal). To simplify the modeling approach, the first assumption of the model is that there is no exchange between axonal and extra-axonal diffusion (despite experimental evidence that suggests exchange is apparent to some extent, see Online Resource 1, section B). In addition, we assume that; free diffusion occurs within the CSF compartment (described as Gaussian diffusion), hindered diffusion (described as diffusion tensor) occurs within the extra-axonal compartment, and restricted diffusion occurs within the intra-axonal compartment (further described by motion within impermeable cylinders, (Van Gelderen, Des Pres, et al., 1994)) (Eq. 1). This is the second assumption of the model, although it is plausible that restricted diffusion also occurs to some extent in the extra-axonal space.

1 $$E(b,\varDelta )={A}_{f}\bullet {E}_{f}+{A}_{h}\bullet {E}_{h}+ {A}_{r}\bullet {E}_{r}$$

Where:

- $E(b,\varDelta )$ is the observed signal decay (Δ is the diffusion time, and $b$ is the diffusion weighting)
- $A$ and $E$ represent the volume factions and signal decays of the three compartments (respectively): free diffusion (*f*), hindered diffusion (*h*), and restricted diffusion (noted by *r*).

In AxSI, common diffusion indices (e.g. mean diffusivity in the hindered space) as well as fiber orientations are estimated separately by conventional procedures (Johansen-Berg & Behrens, 2009). As such, the modeling of ${E}_{f}$ and ${E}_{h}$ is done separately and taken as priors (predictors) for compartment population estimations (see library-based analysis below). Yet, the most complicated and controversial modeling of any axon diameter estimation framework is the restricted diffusion component, which is presumed to represent intra-axonal water. AxSI takes advantage of the *in-vivo* insensitivity of the measured signal to the gradient pulse duration ($\delta$), which is a key factor in modeling strategy decisions (see Online Resource, sections B and C). This insensitivity allows the use of a simple relation between the measured signal and the axon diameter developed previously for certain experimental conditions (Van Gelderen, DesPres, et al., 1994). As the *in-vivo* measured signal is insensitive to the experimental conditions ($\delta$, q-value ; *vide infra*), and its sensitivity to diffusion time reaches a plateau at a certain point (Assaf & Cohen, 1998; Kärger, 1996), the use of Eq. 2 to describe diffusion within impermeable cylinders as a proxy to axons appears adequate and feasible:

2 $${E}_{ax}(q,R)={E}_{0}{e}^{-{q}^{2}{R}^{2}}$$

Where:

- $q$ is the diffusion weighting factor that incorporates the gradient strength and gradient duration ($q=\gamma \delta g$)
- $R$ is the axon diameter

The simplification of the relation between the measured signal and the axon diameter (Eq. 2) dramatically eases the implementation of the framework for axon diameter estimation. Yet, the novelty of AxSI is the estimation of the axonal diameter distribution (ADD) using a linear fit of the measured signal to a set of predictors (library) that simulate, per voxel, the diffusion MRI signal for a series of predetermined axon diameters that cover the range of possible CNS axon diameter values. This axonal spectrum diffusion MRI signal library, which also includes the ${E}_{h}$ and ${E}_{csf}$ estimation, is fitted to the measured signal to estimate the relative weight or contribution of each axonal predictor to the measured signal as well as the population fractions of the hindered and CSF components.

Critical to the suggested fitting routine is a regularization term (in our case Tikhonov approach) that ensures fitting optimization to a smooth axon distribution function, overcoming the possible overfitting to the noisy and limited number of measured signals as given in Eq. 3:

3 $${E}_{r}(b,\varDelta ,R)={\varSigma }_{k=1}^{k}{\pi }_{k}E\left[{E}_{r}\right(b,\varDelta ,{R}_{k}\left)\right]$$

Where ${\pi }_{k}$ are the weights for each predictor.

In this case, we can estimate directly ${\pi }_{k}$ using constrained Tikhonov regularization. Once the weights of different diffusion components are computed (in our implementation there are 160 axon diameters), we can compute several maps including axonal volume fraction, mean axon diameter as well as extra-axonal diffusivity. Using conventional DTI based fiber tracking, we can compute, per reconstructed streamline, the eigenvector corresponding to the highest eigenvalue. This direction is used as the simulated perpendicular direction to the estimated signal decay, per voxel. Later, MSMT-CSD based fiber reconstruction, is used to average axon diameter along a path and is calculated to demonstrate variations in axonal populations across different fascicles (see Methods).

AxSI analysis script is available at: https://github.com/HilaGast/AxSI.git.

## Results

### Axon Diameter Weighted Connectome and Fiber Maps

Using the AxSI framework, we were able to generate three-dimensional brain fiber representations (see Methods) that embed axon diameter information to the extracted fiber tracts.

**Fig. 1:**
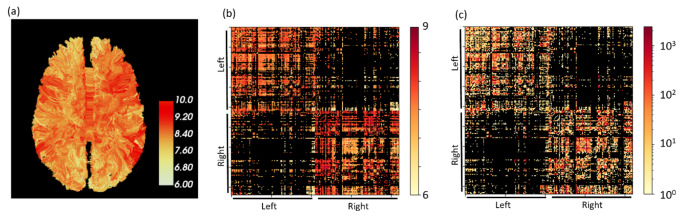
ADD weighted Connectome (**a**) Whole brain tractography of a single subject, weighted by the average ADD. (**b**) Network matrix representation of the same subject, weighted by the average ADD of streamlines that connect each pair of brain regions. (**c**) Network matrix representation of the same subject, weighted by the number of streamlines that connect each pair of brain regions

Figure 1a presents an example of a single subject whole brain representation of streamlines weighted by the average ADD along each streamline. From this representation, network matrices with edges weighted by the average ADD or the number of streamlines was calculated (Fig. 1b and c, respectively). Notably is the difference between the streamlines and ADD weighted connectomes (Fig. 1b-c) highlighting different aspects of network properties.

**Fig. 2:**
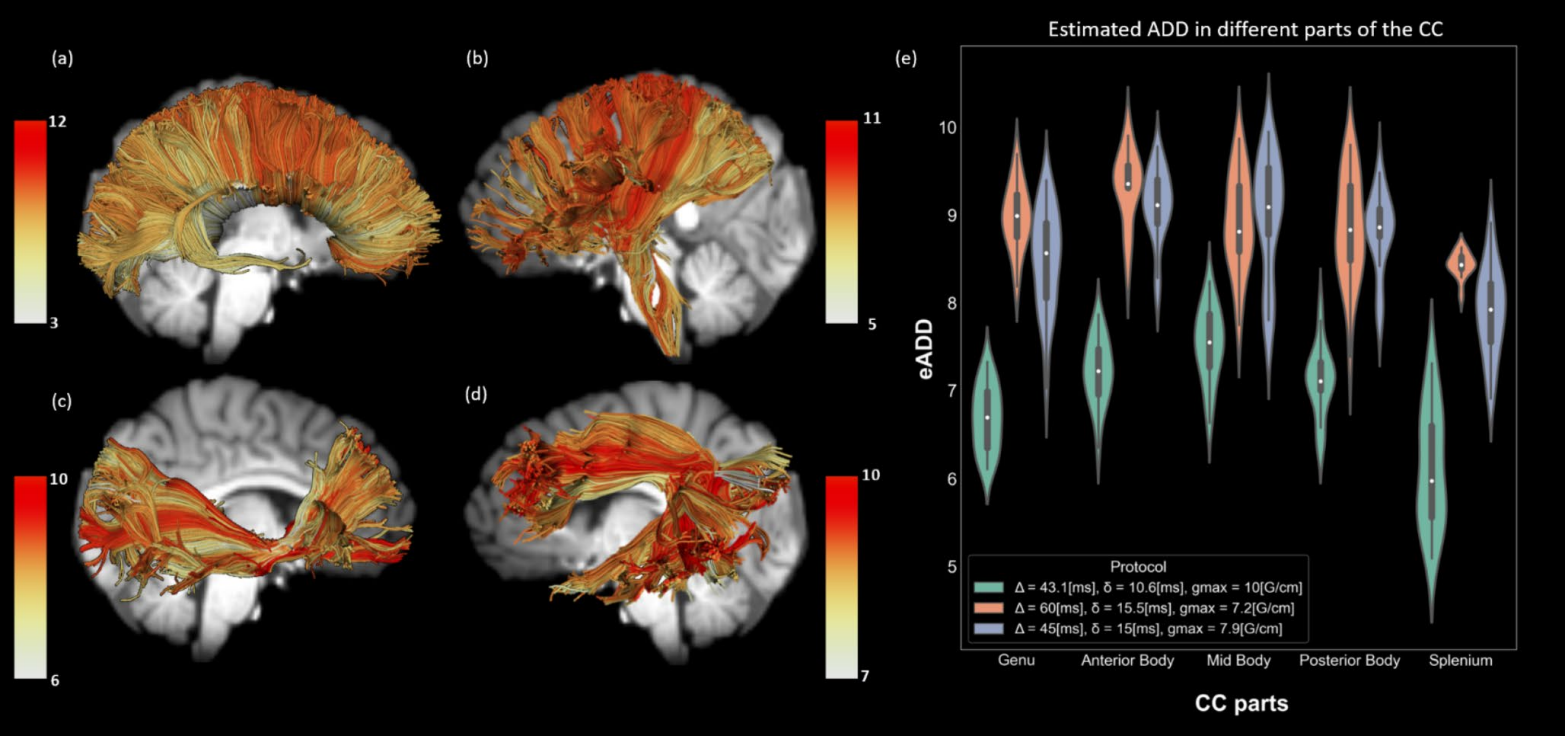
**eMAD values resulting from AxSI analysis** (**a-d**) Visual representations of the eMAD distribution in the CC (**a**), CR (**b**), IFOF (**c**) & SLF (**d**) bundles from AxSI analysis of a single human subject. Different colors represent different estimations of ADD (in $\mu m$, see colorbars). (e) Violin plots for the estimated axon diameter in different parts of the CC, for three different protocols (see Methods). White point represents the median value, mini box for quartiles and the violin is a kernel density estimation of the underlying distribution.

Such maps can visualize and quantify known trends in axon diameter distribution in the brain (e.g., the corpus callosum (Aboitiz et al., 1992)) and other representations that are less commonly described in the literature. In Fig. 2A a representation of the ADD in the CC of a single subject is demonstrated. A violin plot shows the known trend of smaller ADD values in the Genu and Splenium and larger for the Body parts of the CC, using the AxSI framework, for three different diffusion protocols (Fig. 2E, see details in Methods). Repeated-measures ANOVA resulted in significant differences between different parts of the CC for all three protocols: Δ/δ = 43.1/10.6_ms_; F = 19.9, p < < 0.01, Δ/δ = 60/15.5_ms_; F = 4.02, p < 0.01, Δ/δ = 45/15_ms_; F = 15.43, p < < 0.01. Post-hoc analysis of paired differences resulted in significant higher values for body parts compared to Genu or splenium for most comparisons. For example in the HCP protocol (Δ/δ = 43.1/10.6_ms_): t_Anterior Body,Genu_ = 5.14, p < < 0.01, t_Mid Body,Genu_ = 9.53, p < < 0.01, t_Posterior Body,Genu_ = 4.12, p < 0.01, t_Anterior Body,Splenium_ = 6.556, p < < 0.01, t_Mid Body,Splenium_ = 8.00, p < < 0.01, t_Posterior Body,Splenium_ = 5.88, p < < 0.01. All p-values were corrected using Bonferroni correction for multiple comparisons. One protocol (Δ/δ = 60/15.5_ms_) didn’t demonstrate higher values for body parts compared to Genu part. Detailed post-hoc results presented in Online Resource 2, Table S2.

Other tracts also demonstrated patterns and variability in ADD. In Fig. 2a-d, different streamlines are colored according to their estimated mean axon diameter (eMAD), where a ‘hot’ color-scale is used to visualize the different axon diameter (red represents larger axons). It is possible to observe that CST in the middle of CR is salient by its larger axons compared to its surroundings (Fig. 2b) (shown previously in (Huang et al., 2020)). Moreover, both the SLF and IFOF seem to have several sub-bundles, distinguished from each other by their ADD (Fig. 2c-d).

### Axon Diameter Weighted Cortical Surface Representation

**Fig. 3:**
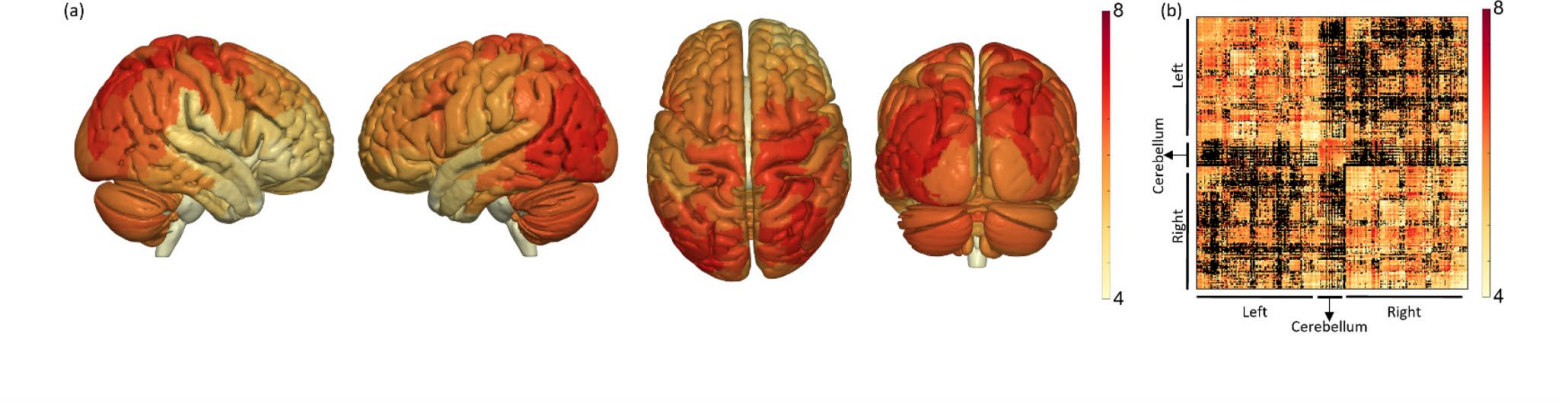
**Axon diameter weighted connectome** (**a**) Cortical surface representation of the average eMAD value of all streamlines connected to each brain region from the Brainnetome Atlas, from four different points of view. Darker color (redder) represents higher values (in $\mu m$, see colorbar). (**b**) Average connectivity matrix of eMAD weighted connectome

A group analysis with AxSI framework on HCP subjects (see Methods), was done to create an axon diameter weighted surface representation and average connectivity matrix. In Fig. 3a, each cortical area from the Brainnetome Atlas is weighted by the average value of eMAD of all streamlines entering/exiting it and averaged again for the entire group. It is easy to notice that each brain region is characterized by different axon sizes that connect it to other parts of the brain. Darker colors (darker red) in the figure represent larger axons. Figure 3b shows the connectivity matrix weighted by the average eMAD over the entire group.

### Averaged Axon Diameter White Matter Reference Map

**Fig. 4:**
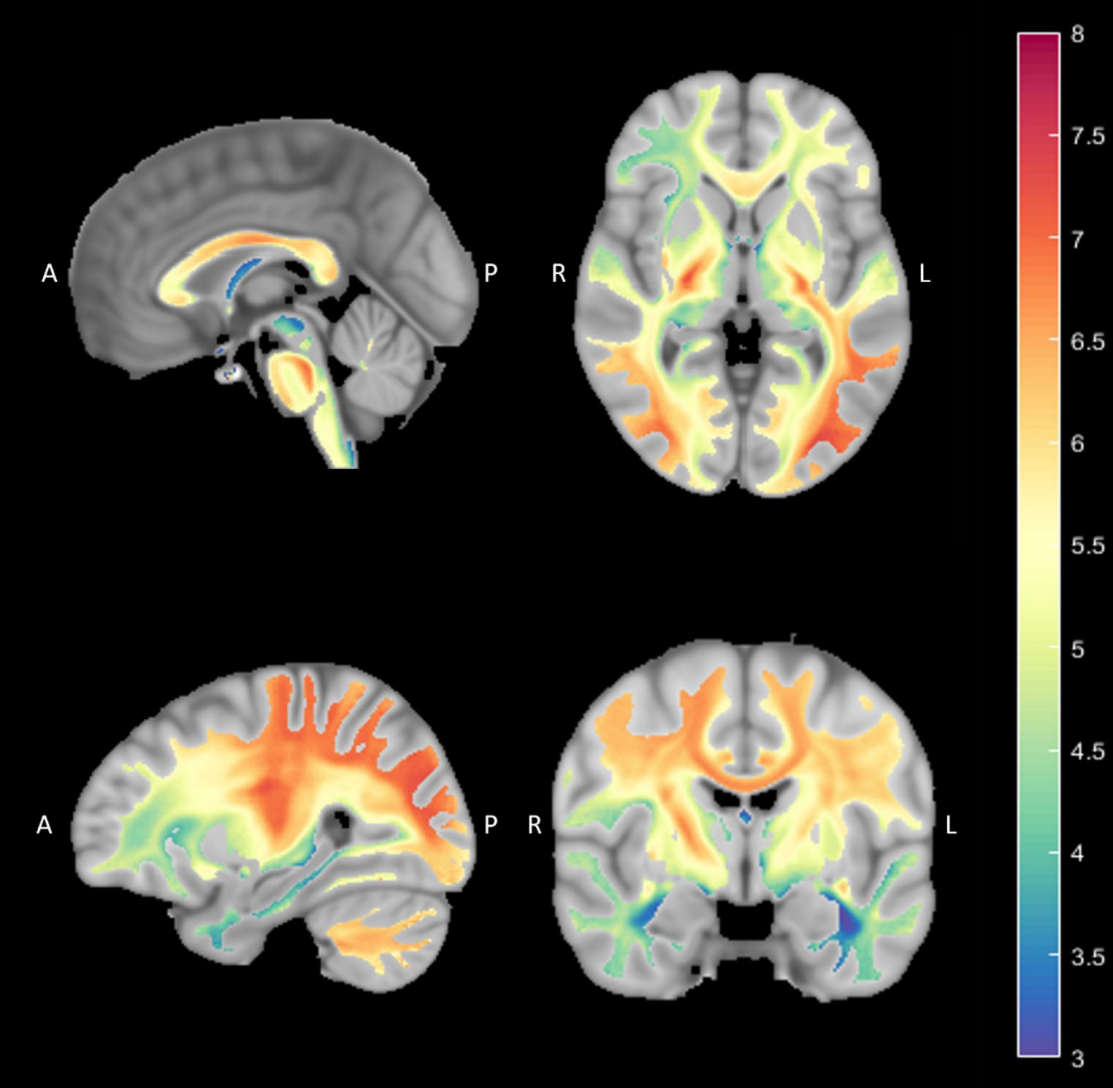
**Average axon diameter WM reference map** Highlight slices from the resulting map demonstrate interesting characteristics of axon diameter distribution in the WM as resulted from a group analysis of HCP data. Colors represent voxel-based averaging eMAD for subjects’ maps after registration to MNI space (in µm, see colorbar)

Finally, we created an average axon diameter WM map, using the group of HCP subjects (see Methods). The maps, created using the AxSI framework, were then registered to MNI space to enable averaging across subjects. The resulting map represents the distribution of estimated axon diameter in the white matter of the healthy human brain young adult. Figure 4 shows highlight slices from this WM atlas (entire dataset is given in Online Resource 2 Fig. S5). For example, in the mid sagittal slice (upper left), the known pattern of ADD in the CC is demonstrated. In a sagittal slice of the left hemisphere (lower left), the CST is salient in red, for having larger axons, as expected. Interesting to note in the axial slice (upper right) the difference in eMAD between the anterior and superior limb of the thalamic radiation. Another pattern worth mentioning appears in the WM of the temporal lobe in the coronal slice (lower right). It demonstrates several different bundles, that differ in their eMAD, with blue\purple that represent lower values, for the Fornix (Fimbria)/Stria Terminalis, green-yellow represent larger values in the ILF (Inferior Longitudinal Fasciculus) and yellow-orange spot for the IFOF with the largest eMAD in this section.

## Discussion

The axon diameter is one of several important measures of the nervous system that provide unique insight into the physiology of information transfer in the brain. While traditionally, axonal morphometry could have been measured only by invasive histological procedures, the suggested framework in this paper, AxSI, offers a platform for estimating axonal properties in-vivo and non-invasively. Over the last decade it has been repeatedly shown that diffusion imaging is sensitive to axonal size (Assaf et al., 2008, 2013, 2019; Barakovic et al., 2021; Barazany et al., 2009; Daducci et al., 2015; Drakesmith et al., 2019; Dyrby et al., 2013; Harkins et al., 2021; Rasclosa et al., 2015; Romascano et al., 2020; Veraart et al., 2020). While the magnitude of this sensitivity is still under debate, it is agreed that certain experimental conditions may favor this unique axonal characterization. Despite the discussion on how to increase the sensitivity of diffusion imaging to axonal properties, the information embedded in axon diameter estimation using MRI is unequivocal.

In this paper we used AxSI to estimate the mean axon diameter property and combined it with traditional fiber tracking to visualize tract-specific axonal properties. Each tract shown in Figs. 1 and 2, was colored according to the mean eMAD of the voxels that contribute to the tract (see Methods). Using this visualization procedure, some known neuroanatomical features of axon fascicles become apparent, consequently increasing the validity and impact of the method. For example, the ability to visualize the pattern of axon diameter changes along the corpus callosum (Fig. 4, top-left panel), highlighting the high eMAD in the body of the CC while smaller values in the splenium and genu region, became the hallmark of axon diameter validation (Aboitiz et al., 1992). Moreover, the higher eMAD values in the cortico-spinal tract compared to other segments of the Corona Radiata imply the fast transmission of signal along the motor pathways compared to other fascicles. Noteworthy is the small axon diameter measured at the frontal/temporal transition zone, where the Uncinate and inferior Fronto-Occipital fascicle passes to the frontal lobe, which is in agreement with histological findings (Liewald et al., 2014).

Comparison of AxSI results with histology is limited. First, there is a very limited number of studies measuring axon diameter properties of different fascicles in the human brain (Aboitiz et al., 1992; Liewald et al., 2014; Olivares et al., 2000). Second, the shrinkage of the tissue in histological preparation underestimates the real axon diameter and probably reduces the variability across fascicles considerably. This stands in contrast to AxSI (and previous methods) that overestimates the axon diameter values. Yet, the above-mentioned observations and comparisons with histology provide sufficient validation to AxSI, thus enabling it to explore other uncharted variations in axonal properties of different tracts. For example, the two massive long-range connections in the human brain: the inferior Fronto-Occipital fasciculus (IFOF) and superior longitudinal fasciculus (SLF) appear to have sub segments with different axon diameter properties (see Fig. 2c-d). At least for the SLF, these segments resemble that anatomical separation of the SLF into 3 segments. Still, the relevance of these observations should be tested in future studies that will try to relate reaction time or other behavioral aspects that should be related to these fiber-systems across a large population cohort.

The surface presentation of AxSI indicates a unique view of the cortex colored by the eMAD of fibers that project to it. It appears that large fiber fascicles project more frequently to somatosensory and motor areas, as well as to visual and auditory cortices, while lower axon diameter projects to more frontal and anterior temporal regions, probably indicating slower transmission of information to these regions. Such a presentation could be the base for connectome analysis integrating axon diameter properties as weights to the edge strength (Fig. 3b). This might provide a more physiological interpretation of the connectome, rather than more spurious measurements such as number of streamline or mean FA (Bassett et al., 2017; Sporns, 2018).

Lastly, we have computed AxSI on 324 random subjects from the HCP database. From these datasets, we were able to create a mean eMAD map in MNI space, providing a reference quantitative map for future studies (see Online Resource 2, Fig. S5). This map allows us to explore anatomically the eMAD property of different areas in the WM of the human brain (computed eMAD map is available at: https://github.com/HilaGast/AxSI.git). Moreover, it might provide the basis of an eMAD-based WM atlas. Such an atlas would define different WM anatomical areas based on their microstructural physiology.

Alongside the fundamental new insight it presents, the ability of AxSI to infer axonal properties relies on several assumptions and modeling approaches that must be well understood before using this method routinely. First, as in any model, the obtained parameters are only estimated and not directly measured. The word ‘estimated’ should be further emphasized, as MRI cannot reach the resolution level that allows visualization of axons, but it infers their existence and size based on the characteristics of water diffusion. This should not weaken the impact or use of the methodology, since most MRI frameworks suffer from the same indirect interpretation problem: functional MRI does not directly measure brain function but rather susceptibility changes following hemodynamic response to brain activity (Toga, 2015), myelin mapping (Glasser & van Essen, 2011) does not measure myelin but rather relaxometry manifestations of myelination, diffusion MRI does not measure diffusion but rather displacement (Johansen-Berg & Behrens, 2009). Following this jargon, AxSI provides a proxy of the axon diameter, and its extracted indices should be indicated as eMAD or estimated axon diameter distribution (eADD).

In addition, while validation of AxSI against the traditional electron microscopy direct measure of axon diameter has been performed, true validation of this measure in-vivo is obviously out of reach. Yet, previous studies have shown that the estimated axon diameters using AxSI fit the expected axonal size variability along the corpus callosum, indicating the sensitivity of this method to known variability (Barazany et al., 2009; Huang et al., 2015, 2020; McNab et al., 2012; Suzuki et al., 2016). Moreover, correlation with physiological measures, such as conduction velocity, also suggest that this measure of axonal diameter is as physiologically relevant as the traditional electron microscopy measures (Horowitz et al., 2015). Those validations suggest that large population studies are essential to explore new features of brain structure/function relations with AxSI.

Since the first demonstration that diffusion MRI is sensitive to axon diameter over 15 years ago (Assaf et al., 2008), significant limitations and concerns regarding the method have been raised. Additionally, the complicated and somewhat ill-posed modeling framework reduced the applicability of the method to the neuroscientific community. As there are only a few in-vivo markers of brain microstructure properties that have direct physiological meaning, the concerns and obstacles of estimating axonal diameter from MRI should be untangled.

Many papers have dealt with the possible effects of exchange, compartmentalization, and experimental conditions (gradient strength, duration) on the parameters computed from the various frameworks for axon diameter measurement (Brabec et al., 2020; Lee et al., 2018, 2020; Nilsson et al., 2013; Novikov et al., 2018; Paquette et al., 2021; Veraart et al., 2020, 2021). As shown in the supplementary material (Online Resource 1, section A, B and C), while these effects are meaningful, they cannot completely diminish the observed sensitivity to axon diameter (Fig. S1-3). Taking advantage of some experimental conditions can even favor axon diameter over other factors. As such, the experimental conditions as described for HCP scan protocol (including high b-value and gradient strength), provide a diffusion MRI signal that has good specificity and sensitivity to axon diameter.

This experimental optimization still requires a robust and simple modeling framework to increase its applicability and impact. In recent years, the use of machine learning procedures to predict and explain measured signals has become more feasible providing new approaches to estimate free parameters of a model from noisy, sub-sampled data (Ma et al., 2013; Tavor et al., 2016; Zhao et al., 2019). AxSI follows this concept and estimates, per voxel, a set of possible signals that represent different axon diameters. Instead of optimizing the axon diameter directly, AxSI regresses the axon diameter dependent signal library to find the best combination of all possible predictors that explains the measured signal while maintaining smooth weighting distribution function over all possible axon diameters. This approach dramatically simplifies the modeling routine and provides a more robust and stable axon diameter estimation approach.

While the AxSI framework coped with most concerns that were raised over the years, it is still not free from limitations. Aside from conventional MRI limitations that include signal to noise and resolution issues that need to be sufficient to achieve accurate eMAD modeling there are additional, more specific to the method, limitations. To achieve high sensitivity towards axon diameter it is required to increase the relative weighting of restricted diffusion water populations (Assaf et al., 2008; Assaf & Cohen, 2000; Barazany et al., 2009; Huang et al., 2015; McNab et al., 2012). Yet, the ability of a diffusion MRI experiment to be sensitive and accurate to restricted diffusion that occurs in a 5 micron and 0.5 micron axon simultaneously depends, in theory, on the experimental conditions (Huang et al., 2015; Veraart et al., 2020). To be sensitive to small-diameter axons there is a need to apply extremely strong diffusion weighting (high-b values) while using the shortest possible period of gradient duration and high amplitude of diffusion gradients (g). There is no magic number for this sensitivity, some simulations suggest that axons with a diameter smaller than 5 microns will be indistinguishable, while others indicate 2 microns as the minimum barrier depending on the experimental conditions (Assaf & Basser, 2005; Assaf & Cohen, 2000; Novikov et al., 2019).

Moreover, the currently applied AxSI analysis, is calculating the estimated ADD based on the primary streamline direction, as determined by DTI analysis. This result in averaging ADD values which are not in the streamline direction (such as in areas of crossing fibers), while calculating the eMAD value per streamline. However, we estimate this effect to be small, or marginal, while averaging values along streamlines for the entire brain. Future development of the AxSI analysis procedure, might include several ADD estimation for each reconstructed direction, with consideration regarding the complexity it adds to the analysis and appropriate means to avoid overfitting.

## Conclusions

The concerns that have been raised over the years regarding the use of diffusion MRI for measuring or estimating axon diameter properties are indeed troubling and hold back the potential uses of this method in neuroscience. All the above mentioned are a result of modeling and simulations and thus, as long as the mathematical description of the diffusion signal is correct, these concerns are valid (Novikov et al., 2018). However, diffusion MRI is a complicated method to be modeled: First, it measures a stochastic phenomenon; the random motion of water molecules, even for the case of water diffusion within a glass requires several assumptions (Felder & Parker, 1985). Second, the effects of membranes as restrictive or semi-permeable barriers are unknown and hence can only be speculated (Brusini et al., 2019; Busza et al., 1992; Lasič et al., 2011). Third, the ground truth for any axon diameter estimation is histology which may considerably differ from in-vivo conditions(Horowitz et al., 2015). This complexity cannot be resolved by including all possible water pools, biophysical properties (e.g., exchange), and experimental conditions.

Yet, despite the validity and significance of the limitations, none of them, to our understanding, can overrule the sensitivity of diffusion MRI, at specific experimental conditions, to axonal morphometry.

AxSI, as described above, was designed to provide an accessible framework for researchers to study the axon diameter property, based on signal modeling, in the scope of the entire human brain and to enable progression of knowledge regarding the structural connectome. As such, we took the simplest modeling approach and achieved anatomically correct results. In this paper, we focused on the challenges of the overfitting issue and the number of free parameters that hold back the progression of study in this field up until now. Therefore, other parameters that might be present in the signal decay were excluded. With that being said, the AxSI framework could be extended in future studies, to include other factors such as tortuosity and dispersion and to be tested by improvement in performances while they are considered.

Estimation of axon diameter properties non-invasively, via diffusion MRI, provides the sole method that infers, indirectly, information transfer speed in the human brain. As such, its importance to connectome analysis is critical. The ability to explain structural connectivity by means of time it takes to information to move from any two points in the brain provides an additional view of this property. It is expected that when incorporating physiological measures (such as axon diameter) to the structural connectome, its correlation with behavior and function will become more significant. With multi-shell diffusion MRI protocols, which became the state-of-the-art in diffusion acquisition, we anticipate that the estimation of axon diameters with simplified analysis platforms such as AxSI will become the method of choice for connectome and structure-function-behavior relation studies.

## Materials and Methods

### Data

60 human subjects were healthy adults scanned as part of the Tel Aviv University (TAU) Strauss Neuroplasticity Brain Bank, and the imaging protocol included additional sequences that were not used in this study. They were scanned using either diffusion protocol 1 (23 subjects: 12 females, age 19–46 years, mean 26.8) or diffusion protocol 2 (37 subjects: 19 females, age 20–73 years, mean 29.7), see details below.

This data was used for estimating the diffusion protocol effect on ADD values along the corpus callosum (CC) (Fig. 2E).

Moreover, in the demonstration of single-subject connectomes, whole-brain tractography and specific bundles weighted by estimated ADD (Figs. 1 and 2A-D), a scan from diffusion protocol 2 was used (Female, 31 years).

Subjects were scanned on a 3T Magnetom Siemens Prisma scanner (Siemens, Erlangen, Germany) with a 64-channel RF coil and a gradient system reaching 80mT/m. The scans include the following sequences:

1. A multi-shell diffusion-weighted imaging (DWI) sequence, with Δ/δ = 60/15.5 or 45/15 [ms] and b-shells of 250, 1000, 3000 & 5000 or 1000, 2000 & 4000 [s/mm^2^] **(**detailed in Table 1).
2. An MPRAGE sequence, with TR/TE = 2400/2.78 [ms] (detailed in Table 2).

For group analysis of ADD (Figs. 3 and 4), we used scans from the HCP database. We used multi-shell DWI scans and high quality T1w structural images data for randomly selected healthy adults (324 subjects: 180 females, age 22–37, mean 28.9) from HCP 1200 young adults release (Essen et al., 2013). A subset (22 subjects: 13 females, age 22–35 years, mean 28.9) was used for analysis of the diffusion protocol effect on ADD values along the CC (Fig. 2E).

HCP Subjects were scanned on a 3T Magnetom Siemens Skyra scanner (Siemens, Erlangen, Germany) with a 128-channel RF coil and customized SC72 gradient system reaching 80mT/m. In this study the following sequences were used:

1. A multi-shell diffusion-weighted imaging (DWI) sequence, with Δ/δ = 43.1/10.6 [ms] and b-shells of 1000, 2000 & 3000 [s/mm^2^] **(**detailed in Table 1).
2. An MPRAGE sequence, with TR/TE = 2400/2.14 [ms] (detailed in Table 2).

Full protocol details available in the HCP reference manual (https://www.humanconnectome.org/storage/app/media/documentation/s1200/HCP_S1200_Release_Reference_Manual.pdf).

All experiments were performed in accordance with the Declaration of Helsinki. The imaging protocol was approved by the institutional review boards of Sheba Medical Centers and Tel Aviv University, where the MRI investigations were performed. All subjects provided signed informed consent before enrollment in the study.

**Table 1:** Experimental parameters for diffusion scans

Protocol	TR/TE [ms]	Bval [s/mm^2^]	#Dir	maxG [G/cm]	Δ/δ [ms]	#Voxels	Resolution [mm^3^]
1	5200/118	0, 250, 1000, 3000 & 5000	88	7.2	60/15.5	120 × 120 × 90	1.7 × 1.7 × 1.7
2	3500/94	0, 1000, 2000 & 4000	186	7.9	45/15	128 × 128 × 88	1.6 × 1.6 × 1.6
HCP	5520/89.5	0, 1000, 2000 & 3000	288	10	43.1/10.6	145 × 145 × 174	1.25 × 1.25 × 1.25

**Table 2:** Experimental parameters for MPRAGE scans

Protocol	TR/TE [ms]	TI [ms]	#Voxels	Resolution [mm^3^]
1 & 2	2400/2.78	1000	224 × 224 × 160	1 × 1 × 1
HCP	2400/2.14	1000	224 × 224 × 180	0.7 × 0.7 × 0.7

### Preprocessing

The TAU Brain Bank scans preprocessing pipeline was done using FSL (Jenkinson et al., 2012) and includes *topup* with reverse phase scans (Andersson et al., 2003) and *eddy* (Andersson & Sotiropoulos, 2016) corrections, as well as registration of MPRAGE to diffusion image using the Functional MRI of the Brain (FMRIB) linear image registration tool (Jenkinson et al., 2002; Jenkinson & Smith, 2001).

The HCP provides minimally preprocessed images (Glasser et al., 2013). This preprocessing pipeline includes intensity normalization across runs, *topup* and *eddy* corrections, gradient nonlinear correction, and registration of MPRAGE to diffusion image using the Functional MRI of the Brain (FMRIB) linear image registration tool boundary-based registration (BRB). The full pipeline is available online (https://github.com/Washington-University/Pipelines).

Furthermore, all HCP scans were registered to MNI-152 T1 template (Collins, D. L., Neelin, P., Peters, T. M., & Evans, 1994) for group analysis in MNI space of the ADD maps, using the FMRIB non-linear image registration tool (*FNIRT*) (J. Andersson et al., 2007; M Jenkinson et al., 2012).

### Diffusion-based Tractography

The Fiber tracking analysis for TAU Brain Bank scans was conducted using a pipeline of deterministic multi-shell multi-tissue CSD (MSMT-CSD) reconstruction (Flavio Dell’Acqua, Luis Lacerda, Marco Catani, 2014; Hansen & Jespersen, 2016; Jeurissen et al., 2014; Tournier et al., 2007) with Continuous Map Criterion (CMC) as a stopping criterion (Girard et al., 2014; Smith et al., 2012) that accounts for partial volumes. To extract the full tractogram, we used the WM + GM brain mask as seeding masks. Tractography was done using the DiPy Library (Garyfallidis et al., 2014). Total number of streamlines varies between subjects.

The Fiber tracking analysis for HCP scans was conducted using the Mrtrix3 software package (Tournier et al., 2019), which uses an MSMT- CSD reconstruction (Dhollander et al., 2016; Jeurissen et al., 2014; Tournier et al., 2019), followed by anatomically constrained deterministic tractography. Analysis was done using the *SD_stream* algorithm in MRtrix3, extracting 4,000,000 streamlines per subject. To extract the full tractogram, we used the entire brain mask as a seeding mask and then filtered the tracts using the MRtrix3 anatomically constrained SIFT algorithm (Smith et al., 2013) so that 40,000 tracts remained extracted for each tractogram.

### Weighting Streamlines

The AxSI framework resulted in a 3D image, while the value in each voxel represents the eMAD resulting from the analysis. After reconstructing streamlines from the entire brain, each streamline was weighted by the average eMAD value of all the voxels it passed through.

### Network Matrices

Based on AxSI analysis results and whole brain tractography, we calculated the weighted network matrices of each subject, while the nodes were defined as the Brainnetome Atlas areas (Fan et al., 2016), which was built upon a connectivity-based parcellation framework. The edges were weighted as the average ADD of all streamlines connected two brain areas, after a non-linear registration of the atlas to the subject’s diffusion scan space.

### Fascicle Extraction

CC bundles were extracted using a 2D midsagittal CC mask for each subject. The masks were created using automatic region-of-interest selection based on FA map intensity and then manually checked and corrected based on the T1 scans where necessary. We then filtered the full brain tracts to only include tracts passing through the corpus callosum mask. The extraction of Corona Radiata (CR), Inferior Fronto-Occipital Fasciculus (IFOF), and Superior Longitudinal Fasciculus (SLF) tracts, has been conducted manually, using ROI masks, for a single subject from the TAU Brain Bank for demonstration.

### Surface Representations of Average ADD

Network matrices weighted by the average ADD were calculated as described above. We then calculated the mean weight of each edge over the entire group of HCP subjects (excluding zeros from calculation) to create a weighted network matrix of the group. We used Median Absolute Deviation (MAD) outlier detection for each edge, in order to exclude extreme values (Leys et al., 2013).

The group matrix was used to calculate the average ADD arriving at each node in the average weighted network. In the surface representation of the mean ADD weighted brain areas, each area value is a representation of the mean value of ADD of streamlines arriving at it in the averaged network.

### Average ADD Maps

To create the average ADD WM maps, we first created an ADD WM map for each subject, by calculating the average ADD values of all streamlines that pass through each voxel. We then registered all maps to MNI space, as described in the preprocessing section. Finally, we calculated the average value for each voxel and masked the resulting map using a WM mask of the MNI template brain. Values of ADD < 0.3 μm were excluded from calculation to reduce the noise derived from very small ADD related signals.

### Statistics

The AxSI analysis values were used to compare the estimated ADD of each CC section for three different scan protocols. Repeated-measures ANOVA was used for comparisons between the five callosal sections which were segmented according to Witelson’s parcellation (Witelson, 1989).

Post-hoc analysis consisted of pairwise t-tests for each combination of CC parts in each protocol separately (total of 10 possible pairs) and p-values were corrected using Bonferroni correction for multiple comparisons.

## Electronic Supplementary Material

Below is the link to the electronic supplementary material.

Supplementary Material 1

Supplementary Material 2

## Data Availability

All code and averaged results are available in the main text or the supplementary materials. All data is available upon reasonable request. Please contact yalabmri@gmail.com with requests.
