# Supplementary material for: A Method for In-Vivo Mapping of Axonal Diameter Distributions in the Human Brain Using Diffusion-Based Axonal Spectrum Imaging (AxSI)
Source: Neuroinformatics. 2023 Apr 10;21(3):469–82. doi: 10.1007/s12021-023-09630-w (PMC10406702; doi:10.1007/s12021-023-09630-w)
Supplement: Supplementary file 1 — Supplementary Material 1 [file 12021_2023_9630_MOESM1_ESM.pdf]

Supplementary Materials for

A method for in-vivo mapping of axonal diameter distributions in the human brain using diffusion-based Axonal Spectrum Imaging (AxSI)

Hila Gast *, Assaf Horowitz, Ronnie Krupnik, Daniel Barazany, Shlomi Lifshits, Shani Ben-Amitay, Yaniv Assaf

*Corresponding author. Email: gast.hila@gmail.com

This PDF file includes:

Supplementary Text
Figs. S1 to S4
Table S1
References

Supplementary Text

Section A. Axon Diameter Specificity and Sensitivity

Several studies have suggested that sources of restricted diffusion, other than intra-axonal space may exist (e.g. in extracellular space) that might compromise the specificity of the signal and even mislead the model estimates (Lee et al., 2018, 2020). Histological analysis of white matter cross section specimens indicates that most of the volume in these areas is occupied by the inner-axonal component (See Figure 1 in (Abdollahzadeh et al., 2019)). While histology might be misleading, since the tissue shrinks dramatically in preparation, especially due to dehydration of the extracellular matrix (Barazany et al., 2009; VIRTANEN et al., 1984), it is still expected that the major part of water signal in diffusion MRI experiments will arise from the intra-axonal compartment. Supporting experimental evidence for this statement comes from water diffusion experiments performed parallel and perpendicular to white matter fascicles, water diffusion experiments on non-myelinated tissue, as well from cortical gray matter area and metabolite (NAA, Choline) diffusion experiments where the extracellular signal is supposedly non-existent (Fig. S1) (Assaf et al., 2002; Assaf & Cohen, 1998, 2000). This phenomenon, published over 15 years ago, indicates that there are multiple populations of water molecules that are distinguishable by their motion properties(Assaf & Cohen, 2000; Beaulieu et al., 1998; Peled et al., 1999). It appears that white matter tissue exhibits multiple water populations in which diffusion is gradually slowed due to motion hindrance (Fig. S1). Particularly is the apparently endlessly slow diffusing component, which is highly anisotropic, originating mainly from intra-cellular signals and affected tremendously by myelination (Fig. S1) (Assaf & Cohen, 2000; Beaulieu, 2002; Stanisz et al., 1997; Stanisz & Henkelman, 1998; Yoshiura et al., 2001).

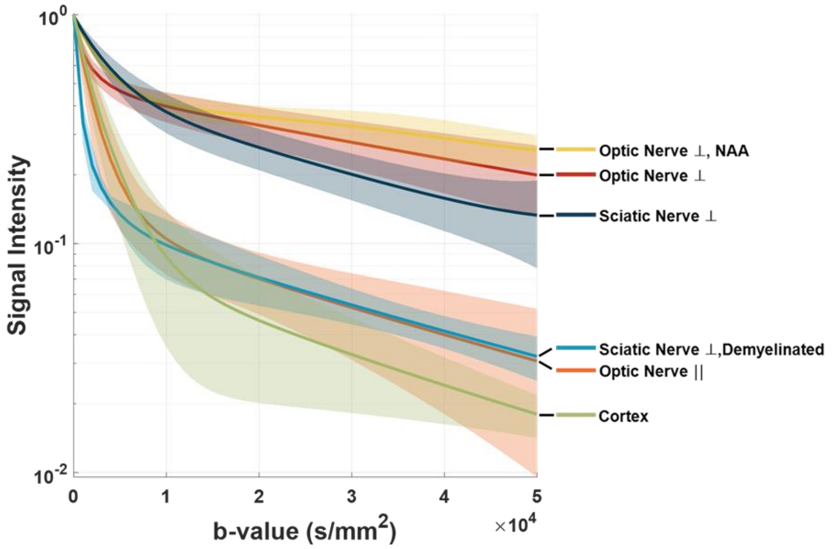

Fig. S1

Water diffusion signal decay for various brain tissue types (cortex, white matter, demyelinated white matter) as well as experimental conditions (measured parallel (‖) or perpendicular (⊥) to fiber's long axis. The data is legacy diffusion MRI data, partially published before (Assaf et al., 2002; Assaf & Cohen, 1998, 2000).

Section B

The limiting parameter that controls the experimental conditions in any diffusion MRI experiment is the amplitude of the diffusion gradients(Huang et al., 2020; McNab et al., 2012). Usually, in diffusion MRI, the protocol sets the diffusion gradients to the maximum value and then calculates the other parameters (diffusion time, Δ, diffusion gradients length, δ). It has been shown, both in theory and through simulations, that the sensitivity towards axon diameter reduces dramatically when the diffusion gradient length becomes longer than a few milliseconds (Assaf et al., 2008; Assaf & Cohen, 2000; Dyrby et al., 2013; Sepehrband et al., 2016). It was suggested that good estimation of axon diameter could be achievable only in preclinical scanners or clinical scanners with very strong gradient systems (such as the connectome magnet) (Lee et al., 2020; Veraart et al., 2020, 2021) or even infeasible at all (Paquette et al., 2021).

However, experimental evidence suggests that the potential implementation obstacles for axonal diameter estimation from diffusion MRI caused by the empirical factor (solving the Bloch-Torrey equations) and hardware considerations (e.g. gradient strength) are not as severe as suspected.

To better understand this, we need to explore the origins of the methodology limitations.

There are three experimental parameters that affect the modeling approach: the diffusion gradient amplitude (g), the diffusion gradient pulse duration (δ) and the diffusion time (Δ). Analytical solutions to the Bloch-Torrey equations for diffusion within cylinders were developed for three experimental conditions: (a) $\delta \ll \Delta$ and $\delta \to 0$ (Ryland & Callaghan, 2010), (b) $\delta < \Delta$ (Van Gelderen et al., 1994) and (c) $\delta \sim \Delta$ (Neuman, 1974).

It has been suggested previously that in-vivo, water exchange between intra- and extra-axonal components, complicates the interpretation of signal decay derived from restricted diffusion (Brabec et al., 2020; Nilsson et al., 2013). Fig. S2 shows data measured in various Δ / δ (see Fig. S3 for examples) combinations of the corpus callosum of the rat brain, in-vivo and ex-vivo according to these approaches (Fig. S2a and S2b). Ex-vivo (Fig. S2b), there is a strong effect of the diffusion gradient duration (δ) indicating that indeed this is a critical parameter in the modeling routine affecting which model to choose (Callaghan, Van-Geldern or Neuman). In-vivo (Fig. S2a), however, the dependency of signal decay on the diffusion gradient duration disappears (in both rodents and humans).

While the invariance of the signal decay to the gradient duration suggests that the modeling could be dramatically simplified (taking a simple approach out of the three mentioned above, for example eq. 14 in (Van Gelderen et al., 1994)), it might also lead to assume that restricted diffusion is minimal in-vivo.

Despite this observation, other experiments suggest that restricted diffusion is significant even in-vivo. In these experiments all conditions are kept similar besides the diffusion time, Δ. Fig. S2c shows variable Δ experiment on the rat corpus callosum measured in-vivo indicating that restricted diffusion affects water motion underscoring the possibility of sensitivity to intra-axonal motion and hence the axon diameter albeit the invariance of the signal to the gradient duration (δ). Moreover, we aimed to separate between the short diffusion time (Δ = 20ms) and the longer ones (Δ>~50ms). As previously shown (Assaf & Cohen, 1998; Kärger, 1996), the effect of the

diffusion time of the signal decay reaches a plateau at the longer diffusion times range indicating that above a certain value it becomes insensitive due to restricted diffusion.

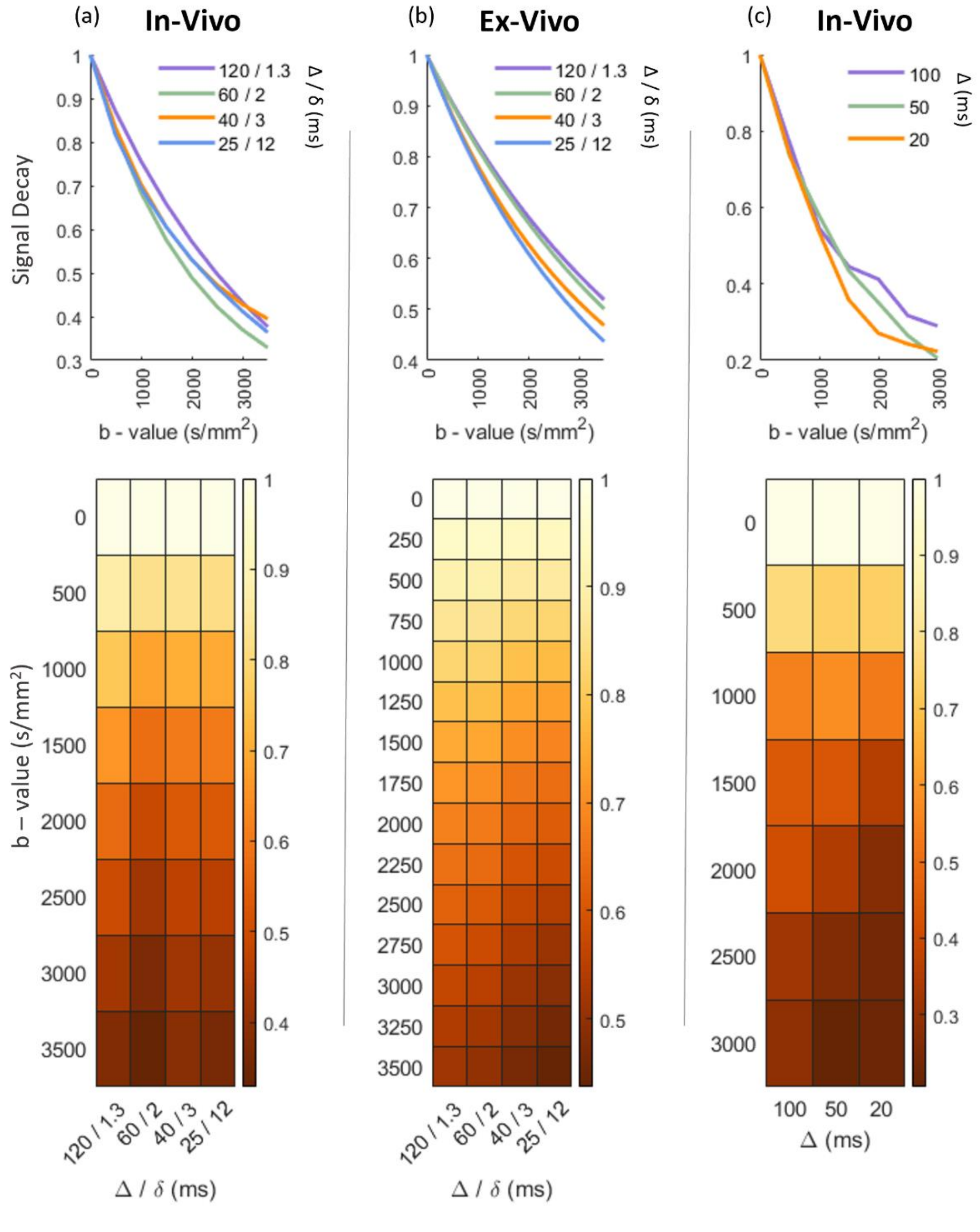

Fig. S2

Controlled parameters experiment for AxSI framework validation. (a-b) Plots (upper panel) and heatmaps (lower panel) of signal decay in the CC of 4 different scan protocols (Experiment 1, 3, 4 & 5 in Table S1), along ascending b-values, for in-vivo (a) and ex-Vivo (b) rat brain scans. (c) A plot (upper panel) and a heatmap (lower panel) of signal decay for experiment with controlled δ and changing Δ, along ascending b-values to examine the ability of the diffusion signal to be sensitive to intra-axonal water motion

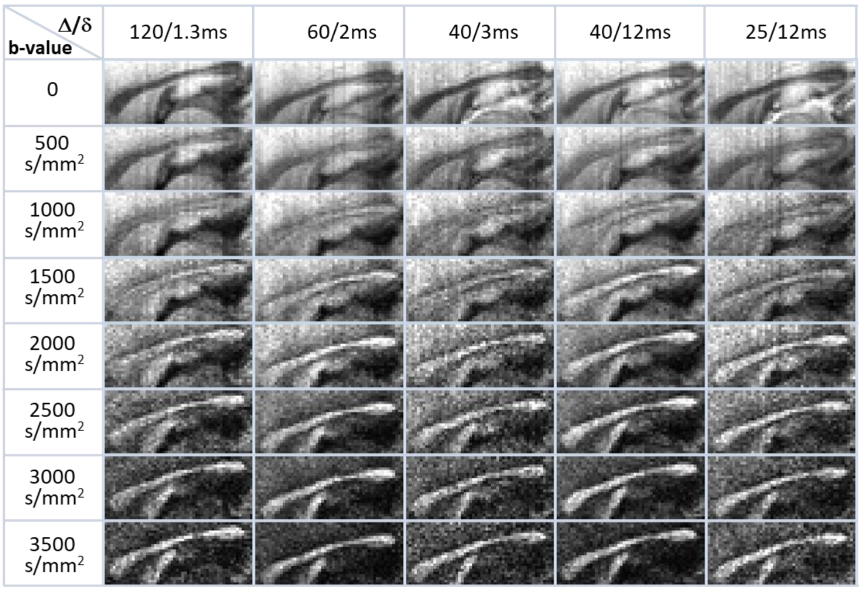

Fig. S3

Examples of in-vivo diffusion MRI signal decay for different experimental conditions at the same b-value range.

Section C: Axon Diameter Estimation in Pre-Clinical Scanner

Clearly, the estimated axon diameter value might deviate from real numbers due to water exchange and other experimental limits (sections A and B) but it can be considered a marker or indicator of intra-axonal diameter. Fig. S4 demonstrates this ability where the axon diameters along the corpus callosum of the rat brain were measured in the most optimal experimental conditions compared with clinical conditions. As indicated from Fig. 4, the in-vivo experimental conditions do not affect the extracted axon diameter trends and appear to linearly shift it towards smaller values. Mantel test for distance comparisons was done to compare the resemblance of the distributions, between each protocol parameters and the optimal protocol (Δ/δ = 120/1.3 $_{ms}$). Resulted correlations were significant for all comparisons. P-value were corrected for multiple comparisons using Bonferroni correction. Results are for the correlation between the mentioned protocol and optimal protocol: $r_{60/2} = 0.63$, $p < 0.01$, $r_{40/3} = 0.57$, $p < 0.01$, $r_{40/12} = 0.52$, $p < 0.01$, $r_{25/12} = 0.51$, $p < 0.01$.

Even though we demonstrate a single sample for each protocol parameters and therefore, no statistical analysis performed to measure it, all experimental conditions resembled the known trend of ADD along the CC (Suzuki et al., 2016).

The above-mentioned experimental drill-down indicates that axon diameter estimation with diffusion MRI is feasible. While absolute measurement of the exact diameter distribution in a region or fiber bundle could be limited due to biophysical

and experimental limitations, the numerical values do represent intra-axonal morphology (Fig. S4).

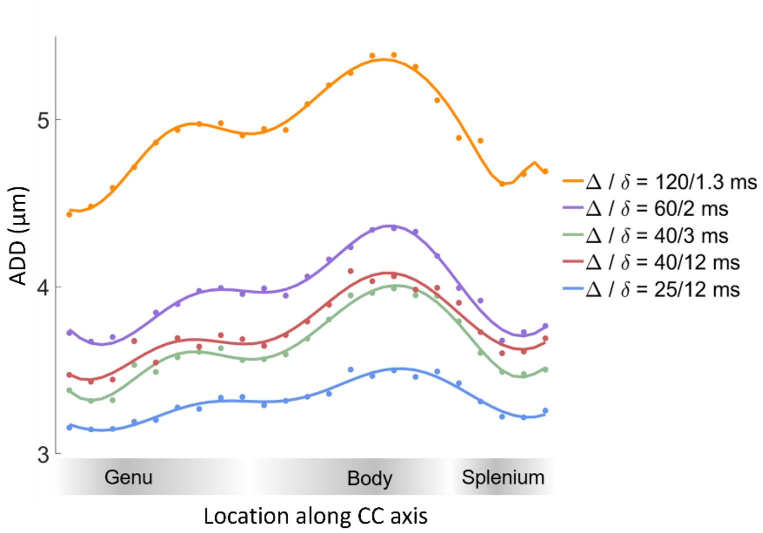

Fig. S4

Mean Axon Diameter Distribution, as resulted from the AxSI analysis for five different protocol parameters, marked with different colors (see Table S1 Experiments 1-5 in Methods). Dot markers represent an average value around a voxel in the skeleton of CC and the solid lines are the corresponding third-degree polynomial functions.

Supplementary methods:

<u>Data:</u>

Rats were scanned at a 7T Bruker 70/30 Biospec system. The imaging protocol consisted of a series of diffusion weighted echo planar images with experimental parameters summarized in Table 1 to control for the effects of gradient pulse duration (δ) and gradient separation (diffusion time, Δ). In all scans the diffusion gradients were applied perpendicular to the corpus callosum.

<u>Measures of ADD distribution along the CC axis:</u>

CC masks for mid-sagittal slices in rat scans were created using automatic region-of-interest selection based on DWI scan intensity. A skeleton was extracted for each scan protocol (Experiment 1-5 in Table S1) and 23 equally spaced points along the skeleton were chosen. Then, each voxel in the CC mask was associated with the closest point to it and the values were averaged to represent the mean value of each of the 23 points. Finally, a third-degree polynomial distribution was matched to each experiment's values.

<u>Comparing along CC distributions:</u>

Mantel test (N Mantel, 1967) was conducted to compare distance matrices between protocols. First, the distance matrix of each protocol was calculated as the difference between each pair of values along the skeleton (as shown in the graph Fig. S4). Then, Mantel test was conducted on each distance matrix with the distance matrix of Δ/δ = 120/1.3 $_{ms}$. The resulted p-values were corrected using Bonferroni correction for the 4 comparisons.

Exp.	TR/TE [ms]	Bval [s/mm^2]	#Dir.	maxG [G/cm]	Δ/δ [ms]	#Voxels	Resolution [mm^3]
1	3000/45	0, 500, 1000, 1500, 2000, 2500, 3000 & 3500	1	7.9	25/12	128x192x8	0.15x0.15x1.5
2	3000/45	0, 500, 1000, 1500, 2000, 2500, 3000 & 3500	1	6.0	40/12	128x192x8	0.15x0.15x1.5
3	3000/45	0, 500, 1000, 1500, 2000, 2500, 3000 & 3500	1	24.8	40/3	128x192x8	0.15x0.15x1.5
4	3000/45	0, 500, 1000, 1500, 2000, 2500, 3000 & 3500	1	30	60/2	128x192x8	0.15x0.15x1.5
5	3000/45	0, 500, 1000, 1500, 2000, 2500, 3000 & 3500	1	33.9	120/1.3	128x192x8	0.15x0.15x1.5
6	1750/18	0, 500, 1000, 1500, 2000, 2500 & 3000	1	33.4	20/3.5	128x192x8	0.15x0.15x1.5
7	1750/18	0, 500, 1000, 1500, 2000, 2500 & 3000	1	20.8	50/3.5	128x192x8	0.15x0.15x1.5
8	1750/18	0, 500, 1000, 1500, 2000, 2500 & 3000	1	14.6	100/3.5	128x192x8	0.15x0.15x1.5

Table S1

Experimental parameters for diffusion scans of Rat experiments.

References:

Abdollahzadeh, A., Belevich, I., Jokitalo, E., Tohka, J., & Sierra, A. (2019). Automated 3D Axonal Morphometry of White Matter. *Scientific Reports*, *9*(1). https://doi.org/10.1038/s41598-019-42648-2

Assaf, Y., Blumenfeld-Katzir, T., Yovel, Y., & Basser, P. J. (2008). AxCaliber: A method for measuring axon diameter distribution from diffusion MRI. *Magnetic Resonance in Medicine*, *59*(6), 1347–1354. https://doi.org/10.1002/mrm.21577

Assaf, Y., & Cohen, Y. (1998). Non-Mono-Exponential Attenuation of Water and N-Acetyl Aspartate Signals Due to Diffusion in Brain Tissue. *Journal of Magnetic Resonance*, *131*(1), 69–85. https://doi.org/10.1006/jmre.1997.1313

Assaf, Y., & Cohen, Y. (2000). Assignment of the water slow-diffusing component in the central nervous system using q-space diffusion MRS: Implications for fiber tract imaging. *Magnetic Resonance in Medicine*, *43*(2), 191–199. https://doi.org/10.1002/(SICI)1522-2594(200002)43:2<191::AID-MRM5>3.0.CO;2-B

Assaf, Y., Kafri, M., Shinar, H., Chapman, J., Korczyn, A. D., Navon, G., & Cohen, Y. (2002). Changes in axonal morphology in experimental autoimmune neuritis as studied by high b-value q-space 1H and 2H DQF diffusion magnetic resonance spectroscopy. *Magnetic Resonance in Medicine*, *48*(1), 71–81. https://doi.org/10.1002/mrm.10183

Barazany, D., Basser, P. J., & Assaf, Y. (2009). In vivo measurement of axon diameter distribution in the corpus callosum of rat brain. *Brain*, *132*(5), 1210–1220. https://doi.org/10.1093/brain/awp042

Beaulieu, C. (2002). The basis of anisotropic water diffusion in the nervous system - A technical review. *NMR in Biomedicine*, *15*(7–8), 435–455. https://doi.org/10.1002/nbm.782

Beaulieu, C., Fenrich, F. R., & Allen, P. S. (1998). Multicomponent water proton transverse relaxation and T2-discriminated water diffusion in myelinated and nonmyelinated nerve. *Magnetic Resonance Imaging*, *16*(10), 1201–1210. https://doi.org/10.1016/S0730-725X(98)00151-9

Brabec, J., Lasič, S., & Nilsson, M. (2020). Time-dependent diffusion in undulating thin fibers: Impact on axon diameter estimation. *NMR in Biomedicine*, *33*(3). https://doi.org/10.1002/nbm.4187

Dyrby, T. B., Søgaard, L. V., Hall, M. G., Ptito, M., & Alexander, D. C. (2013). Contrast and stability of the axon diameter index from microstructure imaging with diffusion MRI. *Magnetic Resonance in Medicine*, *70*(3), 711–721. https://doi.org/10.1002/mrm.24501

Huang, S. Y., Tian, Q., Fan, Q., Witzel, T., Wichtmann, B., McNab, J. A., Daniel Bireley, J., Machado, N., Klawiter, E. C., Mekkaoui, C., Wald, L. L., & Nummenmaa, A. (2020). High-gradient diffusion MRI reveals distinct estimates of axon diameter index within different white matter tracts in the in vivo human brain. *Brain Structure and Function*, *225*(4), 1277–1291. https://doi.org/10.1007/s00429-019-01961-2

Kärger, J. (1996). Diffusion and Perfusion Magnetic Resonance Imaging: Application

to Functional MRI. In *Zeitschrift für Physikalische Chemie* (Vol. 196, Issue 2). Lippincott-Raven Publishers. https://doi.org/10.1524/zpch.1996.196.part_2.278
Lee, H. H., Fieremans, E., & Novikov, D. S. (2018). What dominates the time dependence of diffusion transverse to axons: Intra- or extra-axonal water? *NeuroImage*, *182*, 500–510. https://doi.org/10.1016/j.neuroimage.2017.12.038
Lee, H. H., Papaioannou, A., Novikov, D. S., & Fieremans, E. (2020). In vivo observation and biophysical interpretation of time-dependent diffusion in human cortical gray matter. *NeuroImage*, *222*. https://doi.org/10.1016/j.neuroimage.2020.117054
McNab, J. A., Witzel, T., Bhat, H., Heberlein, K., Keil, B., Cohen-Adad, J., Tisdall, M. D., & Wald, L. L. (2012). In Vivo Human Brain Measurements of Axon Diameter Using 300 mT/m Maximum Gradient Strengths. *Proceedings of the 20th Annual Meeting of ISMRM, Melbourne, Australia*, 7160. https://archive.ismrm.org/2012/3563.html
N Mantel. (1967). The detection of disease clustering and a generalized regression approach. *AACR*220–209 ,*27* ,. https://aacrjournals.org/cancerres/article-abstract/27/2_Part_1/209/476508
Neuman, C. H. (1974). Spin echo of spins diffusing in a bounded medium. *The Journal of Chemical Physics*, *60*, 4508–4511. https://doi.org/10.1063/1.1680931
Nilsson, M., Van Westen, D., Ståhlberg, F., Sundgren, P. C., & Lätt, J. (2013). The role of tissue microstructure and water exchange in biophysical modelling of diffusion in white matter. *Magnetic Resonance Materials in Physics, Biology and Medicine*, *26*(4), 345–370. https://doi.org/10.1007/s10334-013-0371-x
Paquette, M., Eichner, C., Knösche, T. R., & Anwander, A. (2021). Axon Diameter Measurements using Diffusion MRI are Infeasible. *BioRxiv*, 2020.10.01.320507. https://doi.org/10.1101/2020.10.01.320507
Peled, S., Cory, D. G., Raymond, S. A., Kirschner, D. A., & Jolesz, F. A. (1999). Water diffusion, T2, and compartmentation in frog sciatic nerve. *Magnetic Resonance in Medicine*, *42*(5), 911–918. https://doi.org/10.1002/(SICI)1522-2594(199911)42:5<911::AID-MRM11>3.0.CO;2-J
Ryland, B. N., & Callaghan, P. T. (2010). Spin Echo Analysis of Restricted Diffusion under Generalized Gradient Waveforms for Spherical Pores with Relaxivity and Interconnections. *Israel Journal of Chemistry*, *43*(1–2), 1–7. https://doi.org/10.1560/jf3q-url3-5u20-whly
Sepehrband, F., Alexander, D. C., Kurniawan, N. D., Reutens, D. C., & Yang, Z. (2016). Towards higher sensitivity and stability of axon diameter estimation with diffusion-weighted MRI. *NMR in Biomedicine*, *29*(3), 293–308. https://doi.org/10.1002/nbm.3462
Stanisz, G. J., & Henkelman, R. M. (1998). Diffusional anisotropy of T2 components in bovine optic nerve. *Magnetic Resonance in Medicine*, *40*(3), 405–410. https://doi.org/10.1002/mrm.1910400310
Stanisz, G. J., Szafer, A., Wright, G. A., & Henkelman, R. M. (1997). An analytical model of restricted diffusion in bovine optic nerve. *Magnetic Resonance in Medicine*, *37*(1), 103–111. https://doi.org/10.1002/mrm.1910370115
Suzuki, Y., Hori, M., Kamiya, K., Fukunaga, I., Aoki, S., & Van Cauteren, M. (2016). Estimation of the mean axon diameter and intra-axonal space volume fraction of the human corpus callosum: Diffusion q-space imaging with low q-values.

Magnetic Resonance in Medical Sciences, *15*(1), 83–93. https://doi.org/10.2463/mrms.2014-0141

Van Gelderen, P., Des Pres, D., Van Zijl, P. C. M., & Moonen, C. T. W. (1994). Evaluation of Restricted Diffusion in Cylinders. Phosphocreatine in Rabbit Leg Muscle. *Journal of Magnetic Resonance, Series B*, *103*(3), 255–260. https://doi.org/10.1006/jmrb.1994.1038

Veraart, J., Nunes, D., Rudrapatna, U., Fieremans, E., Jones, D. K., Novikov, D. S., & Shemesh, N. (2020). Noninvasive quantification of axon radii using diffusion MRI. *ELife*, *9*. https://doi.org/10.7554/eLife.49855

Veraart, J., Raven, E. P., Edwards, L. J., Weiskopf, N., & Jones, D. K. (2021). The variability of MR axon radii estimates in the human white matter. *Human Brain Mapping*, *42*(7), 2201–2213. https://doi.org/10.1002/hbm.25359

VIRTANEN, J., UUSITALO, H., PALKAMA, A., & KAUFMAN, H. (1984). the Effect of Fixation on Corneal Endothelial Cell Dimensions and Morphology in Scanning Electron Microscopy. *Acta Ophthalmologica*, *62*(4), 577–585. https://doi.org/10.1111/j.1755-3768.1984.tb03970.x

Yoshiura, T., Wu, O., Zaheer, A., Reese, T. G., & Gregory Sorensen, A. (2001). Highly diffusion-sensitized MRI of brain: Dissociation of gray and white matter. *Magnetic Resonance in Medicine*, *45*(5), 734–740. https://doi.org/10.1002/mrm.1100
