# Supplementary material for: A Method for In-Vivo Mapping of Axonal Diameter Distributions in the Human Brain Using Diffusion-Based Axonal Spectrum Imaging (AxSI)
Source: Neuroinformatics. 2023 Apr 10;21(3):469–82. doi: 10.1007/s12021-023-09630-w (PMC10406702; doi:10.1007/s12021-023-09630-w)
Supplement: Supplementary file 2 — Supplementary Material 2 [file 12021_2023_9630_MOESM2_ESM.pdf]

Supplementary Materials for

A method for in-vivo mapping of axonal diameter distributions in the human brain using diffusion-based Axonal Spectrum Imaging (AxSI)

Hila Gast *, Assaf Horowitz, Ronnie Krupnik, Daniel Barazany, Shlomi Lifshits, Shani Ben-Amitay, Yaniv Assaf

*Corresponding author. Email: gast.hila@gmail.com

This PDF file includes:

Table S2
Fig. S5

Protocol Name	CC Parts Pair	t	p-corrected
$\Delta/\delta = 43.1/10.6_{ms}$	Anterior Body, Genu	5.14	<< 0.01
	Anterior Body, Mid Body	-3.07	0.058
	Anterior Body, Posterior Body	1.35	1
	Anterior Body, Splenium	6.56	<< 0.01
	Mid Body, Genu	9.53	<< 0.01
	Posterior Body, Genu	4.12	< 0.01
	Genu, Splenium	2.98	0.070
	Mid Body, Posterior Body	3.84	< 0.01
	Mid Body, Splenium	8.00	<< 0.01
	Posterior Body, Splenium	5.88	<< 0.01
$\Delta/\delta = 45/15_{ms}$	Anterior Body, Genu	7.33	<< 0.01
	Anterior Body, Mid Body	1.37	1
	Anterior Body, Posterior Body	3.48	0.013
	Anterior Body, Splenium	11.3	<< 0.01
	Mid Body, Genu	4.67	<< 0.01
	Posterior Body, Genu	3.23	0.026
	Genu, Splenium	4.89	<< 0.01
	Mid Body, Posterior Body	1.88	0.678
	Mid Body, Splenium	7.46	<< 0.01
	Posterior Body, Splenium	7.40	<< 0.01
$\Delta/\delta = 60/15.5_{ms}$	Anterior Body, Genu	2.87	0.092
	Anterior Body, Mid Body	3.46	0.023
	Anterior Body, Posterior Body	4.20	< 0.01
	Anterior Body, Splenium	9.68	<< 0.01
	Mid Body, Genu	-0.40	1
	Posterior Body, Genu	-0.97	1
	Genu, Splenium	6.33	<< 0.01
	Mid Body, Posterior Body	0.90	1
	Mid Body, Splenium	4.31	< 0.01
	Posterior Body, Splenium	2.99	0.069

Table S2

Pairwise t-test, post hoc analysis for ANOVA results on eADD distribution along the CC, with Bonferroni correction for multiple comparisons.

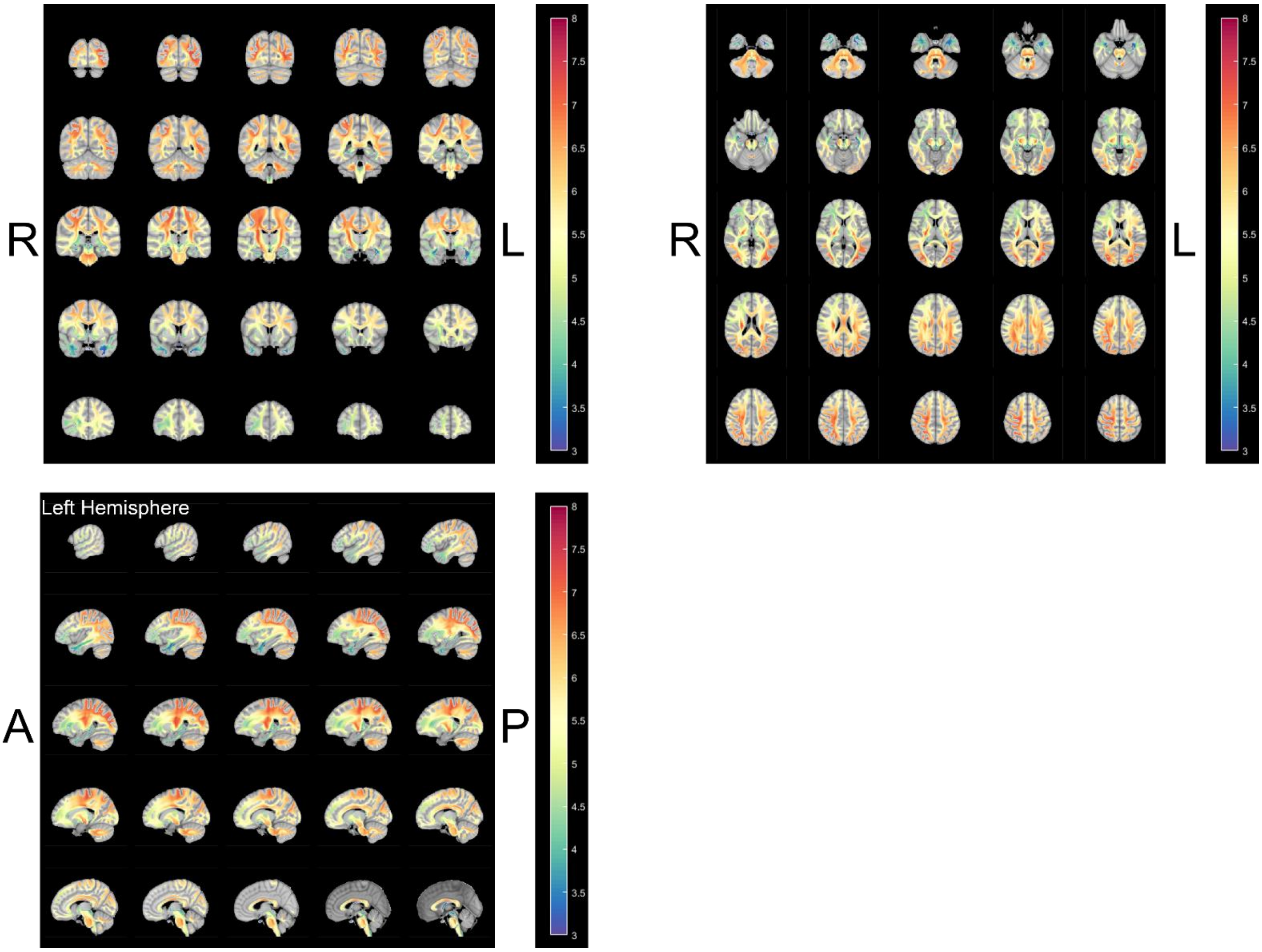

Fig. S5
Average eMAD over a cohort of 324 subjects from the HCP database highlighting the variance of axon diameters across the brain’s white matter. Full map is available at: https://github.com/HilaGast/AxSI.git
